# Oral Lichen Planus as a Late Manifestation of Chikungunya Fever in the Oral Mucosa

**DOI:** 10.1002/ccr3.70499

**Published:** 2025-05-08

**Authors:** Roberta Rayra Martins‐Chaves, Marina Rocha Fonseca Souza, Laura Freitas Xavier, Larissa Rany Martins‐Chaves, Ana Carolina Carneiro Batista de Oliveira, Ricardo Santiago Gomez

**Affiliations:** ^1^ Faculty of Medical Sciences of Minas Gerais Belo Horizonte Brazil; ^2^ Department of Oral Surgery and Pathology School of Dentistry, Universidade Federal de Minas Gerais Belo Horizonte Brazil

**Keywords:** dentistry, dermatology, pathology and laboratory medicine, virology

## Abstract

Chikungunya fever is a viral disease transmitted to humans by mosquitoes. Persistent polyarthritis or polyarthralgia morning stiffness and asthenia may last from serveral months to over three years after the disease onset. A 47‐year‐old non‐smoker was referred for evaluation of multiple bilateral interlacing white plaques and papules associated with erosive areas in the buccal mucosa and the tongue's border and ventral surface. The patient had contracted chikungunya fever ten months earlier and had since experienced ongoing arthralgia and diarrhoea. At the time of the consultation and no dermatological lesions were observed. The microscopic exam of the incisional biopsy was consistent with the diagnosis of interface mucositis. While late mucocutaneous manifestations of chikungunya fever are reported in the literature including the flare‐ups of preexisting dermatological lichen planus and psoriasis the patient was not taking any medication this is the first documented case of oral lichen planus onset associated with this condition.

**Summary**

- While late mucocutaneous manifestations of chikungunya fever have been reported in the literature—including flare‐ups of preexisting dermatological lichen planus and psoriasis—this is, to our knowledge, the first reported case of oral lichen planus onset associated with this condition.

## 1 Introduction

Chikungunya fever is a viral disease transmitted to humans by mosquitoes, primarily *Aedes aegypti* and *Aedes albopictus* , infected with the chikungunya virus. Initial symptoms include fever, myalgia, arthralgia, and skin rash. Constant polyarthritis or polyarthralgia, morning stiffness, and asthenia may persist for months or even longer than 3 years from the onset of the disease [1].

Although early and late manifestations in the oral cavity are reported in patients with chikungunya fever, this is the first documented case of lichen planus as a late manifestation.

## 2 Case History/Examination

**FIGURE 1:**
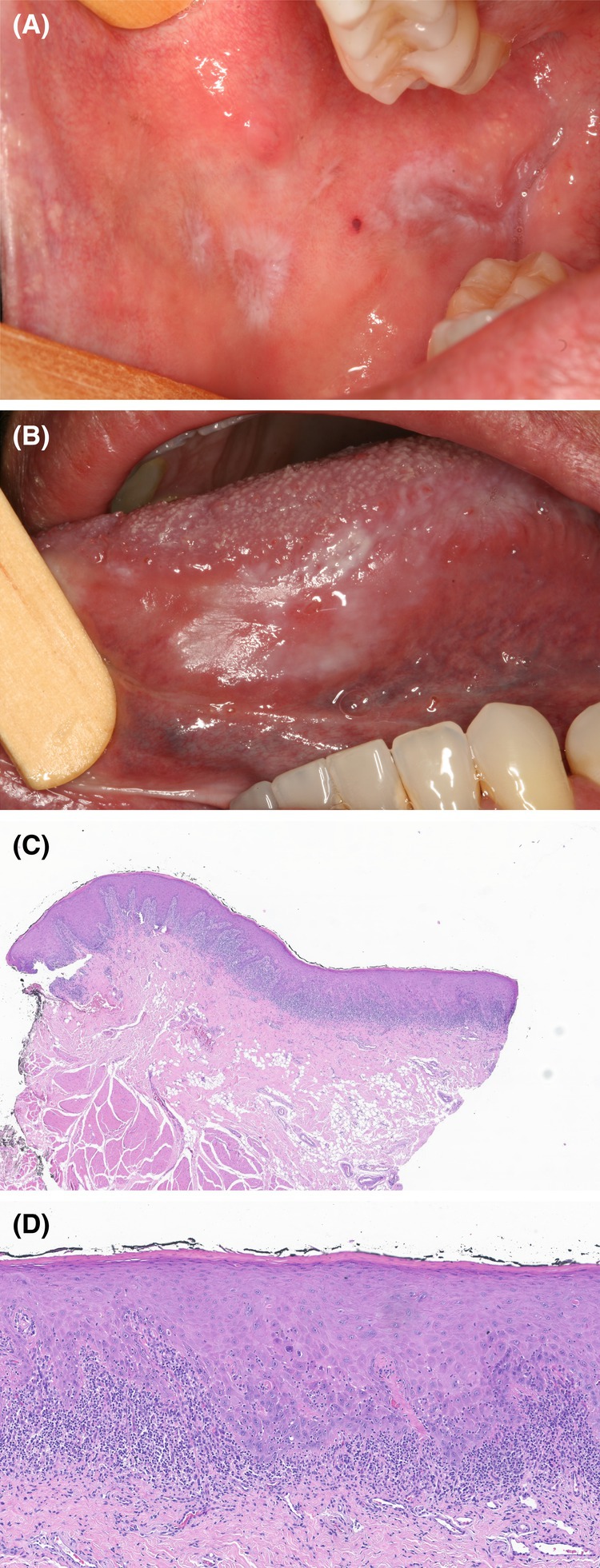
(A) Striated white plaques with central atrophic areas on the buccal mucosa. (B) Plaques with homogeneous and reticular appearance intermingled with erosive areas extending from the lateral border to the ventral surface of the tongue. (C) Incisional biopsy specimen showing a subepithelial band‐like inflammatory infiltrate (original magnification, H&E staining, ×10). (D) Higher‐power photomicrograph exhibiting exocytosis, basal cell layer degeneration, apoptotic bodies and basal membrane hyalinizsation (original magnification, H&E staining, ×40).

A 47‐year‐old non‐smoking man was referred for evaluation of asymptomatic oral lesions persisting for 2 months. The patient had contracted chikungunya fever 10 months earlier, confirmed by RT‐PCR and serological exams, and had since experienced ongoing arthralgia and diarrhea. The main laboratory findings are included in Table 1. At the time of the consultation, the patient reported that the laboratory tests were negative for Chikungunya. Although he had a history of recurrent use of prednisone, tiorfan, and topical nystatin, he was not taking any medication at the time of the consultation. No dermatological lesions were observed. The intraoral evaluation revealed multiple bilateral interlacing white plaques and papules associated with erosive areas in the buccal mucosa and the tongue's border and ventral surface (Figure 1A,B). The patient also reported never having had similar oral lesions.

**TABLE 1:** Main laboratory findings.

Laboratory test	Result
Red blood cells	4,620,000/mm^3^
Hemoglobin	14.1 g/dL
Hematocrit	42%
White blood cells	8490/mm^3^
Neutrophils	4650/mm^3^
Lymphocytes	2430/mm^3^
Monocytes	1330/mm^3^
Eosinophils	60/mm^3^
Basophils	20/mm^3^
Glycated hemoglobin (HbA1c)	5.9%
Estimated average glucose	122 mg/dL
Creatinine	0.89 mg/dL
Rheumatoid fator	3.5 IU/mL
Anti‐HBc IgG	Non‐reactive
Anti‐HCV	Non‐reactive
C‐reactive protein (CRP)	4.0 mg/L
ANA (antinuclear antibodies)	Non‐reactive

## 3 Differential Diagnosis, Investigations, and Treatment

With the provisional diagnosis of oral lichen planus, lichenoid reaction, or another immunomediated condition, An incisional biopsy of the lesion was performed. The histopathological examination showed interface mucositis with degeneration of the basal cell layer, along with a lymphocytic band‐like infiltrate subjacent to the epithelium (Figure 1C,D). Numerous cytoid (Civatte bodies) were also observed (Figure 1D). Since the patient did not develop hypertension, hepatitis C virus infection, or present other possible triggering factors, and was not on any medication at the time of the appointment, the final diagnosis was oral lichen planus as a late manifestation of chikungunya fever.

## 4 Outcome

As the patient denied experiencing pain symptoms, neither systemic nor topical medication was prescribed. After 6 months of follow‐up, no clinical regression was observed.

## 5 Discussion

Previous authors reported early mucosal‐dermatological manifestations of the disease, including vesiculobullous eruptions, Stevens‐Johnson syndrome/toxic epidermal necrolysis‐like eruptions, genital ulcers, maculopapular rashes, pigmentation of the nose, and exacerbation of preexisting dermatoses such as psoriasis and lichen planus [2, 3].

The early oral cavity involvement by ulcers, erythema, oral thrush, gingival bleeding, pain/burning sensations in the oral mucosa, temporomandibular joint arthralgia, dysgeusia, and opportunistic infections were also reported as chikungunya effects [4]. Complete remission usually occurs three to 10 days after the onset of the symptoms [4]. In contrast to acute manifestations, few data in the literature describe the long‐term outcomes of the disease. While late mucocutaneous manifestations of chikungunya fever are reported in the literature, including the flare‐ups of preexisting dermatological lichen planus and psoriasis [2], this is the first report of oral lichen planus onset associated with this condition.

The chronic stage of chikungunya is characterized by an intense influx of inflammatory cells and inflammatory mediators in the joints. A recent study has shown that this may be associated with an imbalanced T regulatory cell response [5]. We hypothesize that a similar mechanism may contribute to the development of oral lichen planus in the oral mucosa.

## Author Contributions

**Roberta Rayra Martins‐Chaves:** conceptualization, formal analysis, investigation, methodology. **Marina Rocha Fonseca Souza:** data curation, formal analysis, investigation. **Laura Freitas Xavier:** formal analysis, investigation. **Larissa Rany Martins‐Chaves:** formal analysis, investigation, methodology. **Ana Carolina Carneiro Batista de Oliveira:** formal analysis, investigation, methodology. **Ricardo Santiago Gomez:** conceptualization, data curation, formal analysis, investigation, supervision, writing – review and editing.

## Consent

Written informed consent was obtained from the patient to publish this report in accordance with the journal's patient consent policy.

## Conflicts of Interest

The authors declare no conflicts of interest.

## Data Availability

The data that support the findings of this study are available in the text of this article.
